# Supplementary material for: Hospital Ownership and Hospital Institutional Change: A Qualitative Study in Guizhou Province, China
Source: Int J Environ Res Public Health. 2019 Apr 24;16(8):1460. doi: 10.3390/ijerph16081460 (PMC6517928; doi:10.3390/ijerph16081460)
Supplement: Supplementary file 1 [file ijerph-16-01460-s001.pdf]

Interview outline to mixed ownership hospitals

1. Please briefly describe the basic situation of your hospital, including affiliation relationship, property structure, governance structure, personnel composition, and operational status.
2. Please introduce the process of the hospital's reform, including the background, motivation and so on.
3. What changes happened in personnel roster, important decision-making, routine management in hospital after reform?
4. What changes happened in how to allocate hospital's revenue and expenditure surplus after reform? Is there executing total wage control like public hospitals?
5. What changes happened in invest sources, competition pressure after reform?
6. What regulations are the government to new mixed ownership hospitals? Is there any difference from before reform?
7. What measures have been done to fulfill social function? Is there any changes compared to before reform?
8. Totally, is there any effects or changes after reform? What are the problems? Do you have any suggestions?

Interview outline to public hospital or private hospital

1. Please briefly describe the basic situation of your hospital, including affiliation relationship, property structure, governance structure, personnel composition, and operational status.
2. At present, what are the main status quo of the hospital in terms of personnel recruitment, hospital decision-making, and daily management?
3. What is the hospital's allocation of income and expenditure balances? Is there a total wage control?
4. What changes happened in invest sources, competition pressure after reform?
5. What regulations and accountability mechanisms are the government to hospital?
6. What measures have been done to fulfill social function?
7. Totally, what are the problems of the hospital? Do you have any plans to solve the problems?

Interview outline to policy makers

1. What are the policy documents for the reform of public hospitals?
2. Where has the reform been implemented in the country? Do you support, oppose or let it develop?
3. What is the top-level design for the supervision and development of public hospitals, private hospitals, and mixed-ownership hospitals?
4. Please briefly explain the distribution of main medical resources this area.
5. Please introduce the process of the hospital's reform, including the background, motivation and so on.
6. What changes have you made to the management and regulation of public hospitals, private hospitals, and mixed-ownership hospitals? Is there any difference among them?
7. Has the new mixed-ownership hospital changed its revenue and expenditure surplus after reform?
8. After the reform, is the government willing to continue to provide financial investment and policy support for new mixed ownership hospitals? If not, do you

think they can survive in hot competition?

9. What regulations and accountability mechanisms are the government to hospital? Is there any difference among public hospitals, private hospitals, and mixed-ownership hospitals?
10. How to evaluate the status quo of these three types of hospitals' operations? Are there any problems? Do you have any suggestions?

Interview outline to researchers

1. How do you think the new mixed-ownership hospitals differ from traditional public hospitals and private hospitals in terms of governance structure and organizational behavior?
2. Based on your research experience, what do you think is the trend of mixed-ownership hospitals? What is the future direction of reform of public hospitals and private hospitals in China?
